# Functional and comparative analysis of *THI1* gene in grasses with a focus on sugarcane

**DOI:** 10.7717/peerj.14973

**Published:** 2023-05-15

**Authors:** Henrique Moura Dias, Andreia Prata Vieira, Erika Maria de Jesus, Nathalia de Setta, Gesiele Barros, Marie-Anne Van Sluys

**Affiliations:** 1 Botanica/IB, Universidade de Sao Paulo, Sao Paulo, Sao Paulo, Brazil; 2 Universidade Federal do ABC, Sao Bernardo do Campo, Sao Paulo, Brazil

**Keywords:** Thiazole biosynthesis, Evolutionary diversity, Genomic characterization, Plant development, Promoter analysis, Genetic complementation, Gene expression

## Abstract

*De novo* synthesis of thiamine (vitamin B1) in plants depends on the action of thiamine thiazole synthase, which synthesizes the thiazole ring, and is encoded by the *THI1* gene. Here, we investigated the evolution and diversity of *THI1* in Poaceae, where C4 and C3 photosynthetic plants co-evolved. An ancestral duplication of THI1 is observed in Panicoideae that remains in many modern monocots, including sugarcane. In addition to the two sugarcane copies (*ScTHI1-1* and *ScTHI1-2*), we identified *ScTHI1-2* alleles showing differences in their sequence, indicating divergence between *ScTHI1-2a* and *ScTHI1-2b*. Such variations are observed only in the Saccharum complex, corroborating the phylogeny. At least five *THI1* genomic environments were found in Poaceae, two in sugarcane, *M. sinensis*, and *S. bicolor*. The *THI1* promoter in Poaceae is highly conserved at 300 bp upstream of the start codon ATG and has cis-regulatory elements that putatively bind to transcription factors associated with development, growth, development and biological rhythms. An experiment set to compare gene expression levels in different tissues across the sugarcane R570 life cycle showed that *ScTHI1-1* was expressed mainly in leaves regardless of age. Furthermore, *ScTHI1* displayed relatively high expression levels in meristem and culm, which varied with the plant age. Finally, yeast complementation studies with THI4-defective strain demonstrate that only *ScTHI1-1* and *ScTHI1-2b* isoforms can partially restore thiamine auxotrophy, albeit at a low frequency. Taken together, the present work supports the existence of multiple origins of THI1 harboring genomic regions in Poaceae with predicted functional redundancy. In addition, it questions the contribution of the levels of the thiazole ring in C4 photosynthetic plant tissues or potentially the relevance of the THI1 protein activity.

## Introduction

The activity of thiamine thiazole synthase (THI1) is key for the biosynthesis of thiamine, also known as a vitamin B1 (Ribeiro et al., 1996). In plants, thiamine is produced through the condensation of two heterocyclic phosphate molecules named hydroxymethyl-thiazole (HET-P) and hydroxymethyl-pyrimidine (HMP-P) (Kong et al., 2008; Jurgenson, Begley & Ealick, 2009; Goyer, 2010; Rapala-Kozik, 2011). As a co-factor of several enzymes, thiamine participates in carbohydrate metabolisms (Rapala-Kozik, 2011; Fitzpatrick & Noordally, 2021) such as pyruvate dehydrogenases involved in the finetuning between glucose and fatty acid oxidation (LeClere, Rampey & Bartel, 2004; Marsden, McMillan & Hanefeld, 2020), transketolase associated with photosynthesis in the Calvin cycle (Frank, Leeper & Luisi, 2007; Rapala-Kozik, 2011; Rocha et al., 2014), and pyruvate decarboxylases that act in alcoholic fermentation (Forlani, Mantelli & Nielsen, 1999). All these pathways and enzymes are essential for maintaining homeostasis and cell functioning.

In addition, to the canonical pathway of the thiazole ring biosynthesis for thiamine production, cumulative evidence in the literature has raised the hypothesis that THI1 plays roles that are still unclear. Initial studies in bacteria and yeast associated *THI1* to tolerance to oxidative stress and DNA repair mechanisms (Machado et al., 1996, 1997; Medina-Silva et al., 2006). In *Arabidopsis thaliana*, THI1 was suggested to have a protective function against salt and osmotic stress linked with the abscisic acid (ABA) hormone signaling (Rapala-Kozik et al., 2012). More recently, Li et al. (2016) have demonstrated that the AtTHI1 protein interacts with Ca^2+^-dependent protein kinases and modulates stomata closure through ABA signaling during drought stress. A single gene encodes the AtTHI1 protein (Machado et al., 1996) which has two signal peptides at the N-terminus of the protein due to the presence of two initiation codons that targets each of the isoforms to either chloroplasts or mitochondria (Chabregas et al., 2001, 2003).

As drought stress strongly constrains crop yield (Dietz, Zörb & Geilfus, 2021), we aimed to understand the evolution and diversity of *THI1* in Poaceae, where C4 and C3 photosynthetic plants co-evolved. Species of agricultural interest, such as rice, corn, wheat, sorghum, and sugarcane, are all members of this important family. Sugarcane (*Saccharum* spp.) attracts special attention due to its ability to accumulate higher levels of sucrose in the stalk internodes (Whittaker & Botha, 1997). Sucrose is the cheapest and one of the most accessible sources of carbon for human and livestock consumption, and sugarcane provides the best ratio of sugar yield per cultivated area worldwide (Formann et al., 2020). Sugarcane is a C4 high-yield photosynthetic crop with the potential for bioenergy. This energy source is explored in Latin America in Brazil, Mexico, and Colombia. Yet, other countries could also increase the contribution of sugarcane to their fuel and energy sectors (Khan & Khan, 2019).

The complexity of the sugarcane genome has hindered its full sequencing and accurate gene annotation. A steady and progressive effort worldwide has produced encouraged several initiatives starting with an EST database for sugarcane (Vettore et al., 2003) and subsequently, a sugarcane BAC collection (de Setta et al., 2014), a monoploid version (Garsmeur et al., 2018) and an allele resolved version of a commercial cultivar (Souza et al., 2019). In all these genomic resources, two *AtTHI1* homologs are identified. Our synteny studies revealed that C4 photosynthetic plants maintain multiple copies of the THI1 gene however with unequal distribution of genomic loci across closely related species. *THI1 g*ene sequence diversification using phylogeny in Poaceae and clustering in Saccharum complex is revealed. *Cis*-regulatory elements in the promoter region, as well as differential gene expression pattern of the gene copies along development support that all three variants are expressed. Furthermore, *ScTHI1-1* and *ScTHI1-2b* were capable of genetically complementing yeast THI4 mutants. Taken together, our findings provide information on the evolution and divergence of the *THI1* gene in Poaceae and shed light on biological aspects of the *ScTHI1* genes in sugarcane.

## Materials and Methods

### Screening of sugarcane *ScTHI1* homologs in the BAC library and in other grasses sequence collection of *THI1* genes in grasses

Based on a conserved region of the two SAS sequences from the SUCEST database (Vettore et al., 2001) identified as putative *THI1* homologs, a pair of primers (thi1_F: CAC CAT GGC CGA GAA CAG; thi1_R: CGT ACG AGC TCT CCA AGG AC) was used to screen for the presence of *ScTHI1* in the sugarcane BAC library (de Setta et al., 2014). As a result, nineteen BACs were selected for sequencing and assembly as described previously (de Setta et al., 2014).

Phytozome database v13 (Goodstein et al., 2012) and PLAZA Monocots 4.5 database (Van Bel et al., 2018) were screened using BLAST search to identify *THI1* homologs in Poaceae group plants with an *E*-value cutoff of 10^−5^ and coverage ≥70%. Only nucleotide and protein sequences from species with complete sequenced genomes were selected. Manual inspection was performed to verify all putative candidates using the online software InterProScan (Blum et al., 2021) and Pfam (El-Gebali et al., 2019) to check the presence of the full THI4 protein family domain (PF01946).

### Phylogenetic and synteny analyses

Full-length protein sequences were aligned and inspected using MAFFT v7.450 (Katoh & Standley, 2013). The topology of phylogenetic tree was generated using the Bayesian analysis algorithm with MrBayes v3.2.6 (Ronquist et al., 2012). The node’s significance was evaluated by one run of 1,000,000 generations with Metropolis-coupled Monte Carlo Markov chains (MCMC). The Bayesian model parameters were nucmodel = 4by4, nst = 2, and aamodel = mixed for amino acid alignments. Markov chains were sampled every 1,000,000 generations. The remaining trees were used to compute the majority rule consensus tree, the posterior probability of clades and branches lengths. Both analyses were performed in Geneious Prime (version 2021.0.3).

To evaluate synteny across species within *THI1* gene region, a 200 kb region was explored for conserved genes both upstream and downstream within the genome of 24 examined species. *A. thaliana* genome sequence was used as the reference, since it is currently the best genome assembly with highest quality and the completest genome annotation. tBLASTn and the best-fit results (E-value ≤ 2e−10 and identity ≥80%) were selected to explore these genome assemblies. An R package was used to design genes in a chromosome-scale (Anand & Rodriguez Lopez, 2022) with adjustments in Inkscape Illustrator.

### *Saccharum spp. THI1* sequence diversity analysis

A network analysis approach was used to address the THI1 homologs sequence diversity in the *Saccharum* complex. A pair of primers (thi1Conserved_F: CTC CTC AAG TCC TCC TTC GC and thi1Conseved_R: TCA TGC CGA TGT CCT GGA G) was used to generate sequences corresponding to a conserved region of *ScTHI1*. Genomic DNA from closely related species *S. spontaneum* (Mandalay), *S. spontaneum* (IN84-58), *Miscanthus sp*.,*S. officinarum*, Brazilian hybrids SP8032-80, SP7011-43, SP8132-50, RB835486, RB72454, RB867515, and other cultivars (POJ-2878 from Java; NA56-79 from Argentina; NCo-310 from South Africa; and Co-290 from India) were used as template in the PCR as well as the R570 nineteen BACs. Amplicons were cloned and sequenced using the ABI PRIS 3730 DNA ANALYZER (Applied Biosystems^™^, Waltham, MA, USA). Sequence quality and assembly was performed with Phred-Phrap-Consed package (Ewing & Green, 1998; Gordon, Abajian & Green, 1998; Gordon, 2003). Only bases with phred quality ≥20 were used, resulting in primary sequences of about 330 bp. Homologous regions from SbTHI1, ZmTHI1, MsTHI1 were retrieved by BLASTn from Phytozome v13 database (Goodstein et al., 2012).

As described in Vieira (2018), ClustalOmega (Sievers et al., 2011) using default parameters was used to align all sequences. DnaSP5 (Librado & Rozas, 2009) and NETWORK 4.6.1.2 (Bandelt, Forster & Röhl, 1999) software with default parameters were used to generate the network graph. Representative sequences from three R570 BACs were selected as follows, 108_C04 BAC represents *ScTHI1-1*, the one found in the 017_B18 BAC represents *ScTHI1-2a* and the 094_O04 BAC represents *ScTHI1-2b*.

### Promoter characterization and distribution of *cis*-regulatory elements in *THI1* homologs

We performed a comparative analysis of the promoter regions of the different occurrences of *THI1* in: *Zea mays*, *Miscanthus sinensis*, *Sorghum bicolor*, *Saccharum spontaneum* (monoploid), and *Saccharum* sp. var. R570. The *THI1* gene sequences were identified *via* tBLASTn on PLAZA database and in BACs of sugarcane (R570 variety), by using the homolog protein sequence of *A. thaliana*, as a query. Upstream sequences of 2,000 bp from the start codon were assessed for conserved features and motifs, by using MEME suite tools (Bailey et al., 2009). Twenty motifs were allowed to be from 5 to 25 bp in length, with an *E-value* less than 0.05, the default parameter for MEME (Powell et al., 2019). The retrieved motifs were run through TomTom (Gupta et al., 2007), *via* the JASPAR Core Plants database (Khan et al., 2018), and the respective Uniprot IDs results were collected, if *p-value* was equal or smaller than 0.01. The Uniprot IDs were then used to collect biological GO terms for functions assignment of each motif, and g:Profiler (Raudvere et al., 2019) was used for statistical analysis of GO terms overrepresentation.

### Subcellular localization prediction

According to Chabregas et al. (2003), we identified two start codons in the *THI1* sequences. The whole amino acids sequence of *THI1* starting from the first start codon (1st ATG) and second (2nd ATG) was used for prediction of signal peptide cleavage site with SignalP (Armenteros et al., 2019b). To subcellular localization prediction was used TargetP (Armenteros et al., 2019a).

### Plant growth conditions for evaluating the expression of *ScTHI1* homologs

Sugarcane (*Saccharum sp*. var. R570) was vegetatively propagated from the GaTE-Lab sugarcane collection (Instituto de Biociências, USP). The culms were disinfected with 1.5% hypochlorite, germinated on vermiculite, and maintained in a greenhouse for 15 days. The seedlings were then transferred to pots (50 L) with a mixture of substrate 3:1 (commercial substrate and vermiculite). Irrigation occurred systematically, with nutrient supplementation 15 days before harvest. Plants were harvested at 3, 6, and 9 months after sprouting, and separated into apical meristem, leaf, culm, and root.

### Total RNA and cDNA synthesis

Total RNA was isolated from fine powder ground tissue with TRIzol (Thermo Fisher Scientific, Waltham, MA, USA) according to the manufacturer’s recommendations. RNA quantity and purity were measured in an ND-1000 NanoDrop spectrophotometer (Thermo Fisher Scientific, Waltham, MA, USA). The quality and integrity of the RNA were verified by electrophoresis on 1% agarose gels. Total RNA samples were treated with a Turbo DNA-*free* kit (Invitrogen, Waltham, MA, USA). Super Script First III Strand System for RT-PCR kit (Invitrogen, Waltham, MA, USA) was used for complementary DNA (cDNA) synthesis from 500 ng/μL RNA samples.

### RT-qPCR assay and gene quantification

Each cDNA sample was subjected to reverse transcriptase quantitative PCR (RT-qPCR) reactions for all genes of interest in each using cDNA-specific TaqManGene Expression Assays on QuantStudio 7 Flex Real-Time PCR System (Applied Biosystems, Waltham, MA, USA). Reaction mixture in a total 10 μl reaction is composed of 1 μl cDNA, 0.25 μL (0.25 μM) TaqMan Probe, 0.3 μL (0.3 μM) of Forward and Reverse primers, 5 μl TaqMan Fast Advanced Master Mix 2x (Applied Biosystems, Waltham, MA, USA) and 3.15 μL nuclease free water. This allowed for the consistent use of standardized thermal cycling conditions: 95 °C for 2 min, followed by 40 cycles of 95 °C for 1 s and 60 °C for 20 s. The standard curve was generated using synthetic genes cloned in BlueHeron pUC MinusMCS plasmid. The standard curve was generated using synthetic CDS of *ScTHI1* genes cloned in BlueHeron pUC MinusMCS vector, serially diluted (10×). The transcripts copy numbers were determined by interpolation of the standard curve. Each sample and standard curve was run in triplicate to ensure reproducibility. Absolute expression data (number of transcripts) were log-transformed to enable statistical analysis (ANOVA), assuming the log normality of the data.

### Yeast complementation assay

As previously described in Vieira (2018), three synthetic versions of *ScTHI1* were performed by Blue Heron^®^ Biotech, LLC. Alternative versions of these CDS lacking the N-terminal chloroplast transit peptide (DelN) were produced through PCR amplification on the synthetic CDSs, using a pair of primers (thi1.1DelN_F: GGA TCC ATG ACC CGC CGC TA for *ScTHI1-1*; and thi1.2DelN_F: GGA TCC ATG ACC CGG CGG TA for both *ScTHI1-2a* and *ScTHI1-2b*) and thi1DelN_R (GTC GAC TCA GGC GTC CAC) for all corresponding to a transcript that includes the second initiation codon (amino acid 76 in Fig. S2) up to the stop codon (amino acid 360).

A restriction enzyme BamH1-Sal1 fragment of the corresponding CDSs were inserted into the yeast expression vector pG-1 (Schena, Picard & Yamamoto, 1991) using T4 ligase (Promega, Madison, WI, USA) according to the manufacturer’s protocol. Two other constructs were used as controls: the positive control A184V, which corresponds to the *A. thaliana tz-201* mutant CDS and is able to complement the thiamine auxotrophy in the *thi4* yeast mutant (Papini-Terzi et al., 2003); and DelN as negative control, which is the *A. thaliana* wild-type (WT) *AtTHI1* lacking the N-terminal chloroplast transit peptide (Papini-Terzi et al., 2003). All constructs were transformed into wild type strain W303 (mata, ade2-1, trp1-1, leu2-3-112, can1-100, ura3-1, his3-11-115) as a positive control (Praekelt, Byrne & Meacock, 1994) and the thiamine auxotroph KBY5 mutant strain (THI4::URA3) by the heat shock method (Schiestl & Gietz, 1989).

All transformant selection was on yeast nitrogen base (YNB) (BD Biosciences, Franklin Lakes, NJ, USA) medium lacking tryptophan. Thiamine auxotrophy was examined by growing cells overnight in liquid YNB without tryptophan, centrifuged, and re-suspended in 10 mM MgSO_4_. From an OD = 1, three serial and tenfold dilutions were prepared. Yeast complementation was assayed on minimal medium (YNB lacking both thiamine-HCL (YNB-thia) and tryptophan, from USBiological) plates, either with or without thiamine and tryptophan, incubated for 28 days at 30 °C, and analyzed every 4 days.

## Results

### Gene synteny of *THI1* homologs in sugarcane and Poaceae

As many Poaceae genomes are publicly available, we analyzed the *THI1* by a comparative genomic approach across a wide range of species. Sequences from *Arabidopsis thaliana* and *Joinvillea ascendens* were included as outgroups. For sugarcane, besides the genome from *S. spontaneum* (Zhang et al., 2018), additional 19 bacterial artificial chromosomes (BACs) (Table S1) from the R570 cultivar (de Setta et al., 2014) containing *THI1* homologs were sequenced and annotated. All sequences containing the putative protein were compared using PFAM and InterproScan databases to screen for the presence of the THI4 protein family domain (PF01946). After removing sequences with incomplete domains and redundant sequences retrieved from this search, 49 THI1 homologs were identified in 23 Poaceae species (Table S2).

**Figure 1:**
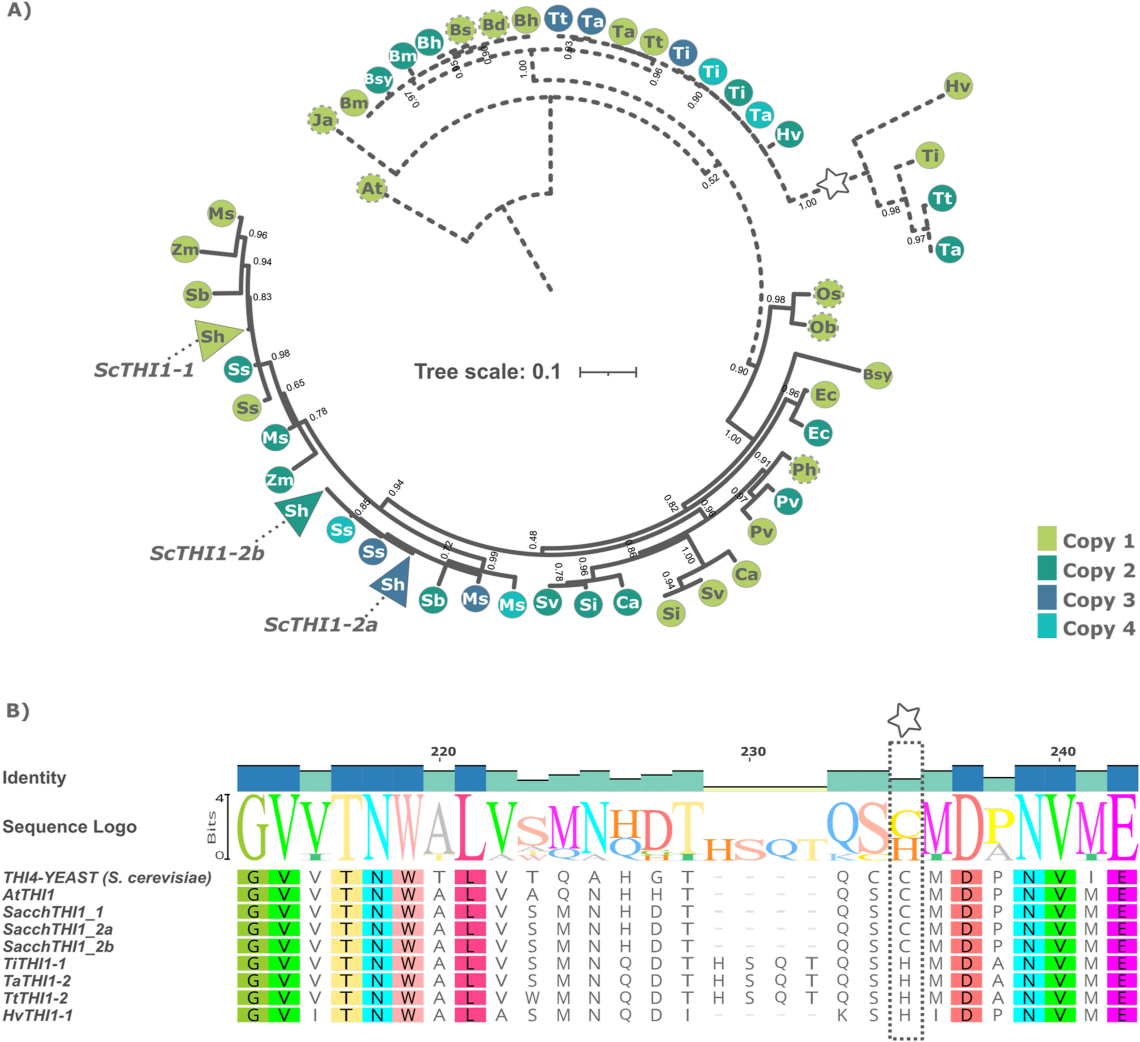
Phylogenetic relationships of THI1 (thiamine thiazole synthase) in Poaceae. (A) The tree containing 51 protein sequences is subdivided into two major clades. Clade A (dashed line) comprises the C3 grasses (*B. distachyum* (Bd), *B. hybridum* (Bh), *B. mexicanum* (Bd), *B. stacei* (Bs), *B. sylvaticum* (Bsy), *H. vulgare* (Hv), *T. aestivum* (Ta), *T. turgidum* (Tt), *T. intermedium* (Ti)) and Clade B (black line) the C4 grasses (*E. coracana* (Ec), *C. americanus* (Ca), *P. hallii* (Ph), *P. virgatum* (Pv), *M. sinensis* (Ms), *Saccharum sp*. var. R570 (*ScTHI1*), *S. spontaneum* (Ss), *S. italica* (Si), *S. viridis* (Sv), *S. bicolor* (Sb), and *Z. mays* (Zm)). The outgroups are represented by *A. thaliana* (At) and *J. ascendens* (Ja). The circle indicate species and are colored according to the numbers of gene copies. Circles with dashed border show a THI1 homolog single copy. The triangle shows collapsed BAC sequences. The scale shows the phylogenetic distance between protein sequences. For an expanded version with protein names and IDs see Table S2. (B) Multiple sequence alignment of THI1 homologs from *Saccharomyces cerevisiae*, *Arabidopsis thaliana*, *Saccharum sp*. var. R570, *Hordeum vulgare*, *Triticum aestivum, Triticum turgidum, and Thinopyrum intermedium* showing diversity at position 238 (stars).

A Bayesian phylogenetic tree based on multiple alignments from these 49 protein sequences and the two outgroups was built. The tree topology revealed that the THI1 homologs were separated into two distinct clades, Clade A and Clade B (Fig. 1A and Table S2). Clade A is composed by THI1-homologue single copy sequences from *B. distachyum*, *B. hybridum*, *B. mexicanum*, *B. stacei*, *B. sylvaticum*, *H. vulgare*, *T. aestivum*, *T. turgidum*, *T. intermedium*. It also displays a diversifying branch from *Hordeum vulgare*, *Triticum aestivum*, *Triticum turgidum*, and *Thinopyrum intermedium* that has a previously described amino acid substitution at position 238 (Fig. 1B and Table S2). The *in-silico* analyses of the R570 sugarcane BAC sequences revealed that there are at least two gene variants of *ScTHI1* supporting the two SAS previously described. Those, alongside with *S. spontaneum* sequences, are distributed into two genomic subgroups with a common ancestral node. The amino acid sequence from ScTHI1 copies derived from the SAS1 probe (ScTHI1-1) clustered with SbTHI1-1, ZmTHI1-1, ZmTHI1-2, MsTHI1-1, MsTHI1-2, SsTHI1-1, and SsTHI1-2, while the sequences derived from SAS2 (ScTHI1-2a and ScTHI1-2b) clustered with SbTHI1-2, MsTHI1-3, MsTHI1-4, SsTHI1-3, and SsTHI1-4. Clade B (*Cenchrus americanus, Eleusine coracana, Miscanthus sinensis, Panicum hallii, Panicum virgatum, Saccharum sp., Setaria italica, Setaria viridis, Sorghum bicolor*, and *Zea mays*) present at least two THI1-homologues, with the exception of *Oryza sativa* and *Oryza brachyantha* that have a single copy THI1 homologue.

**Figure 2:**
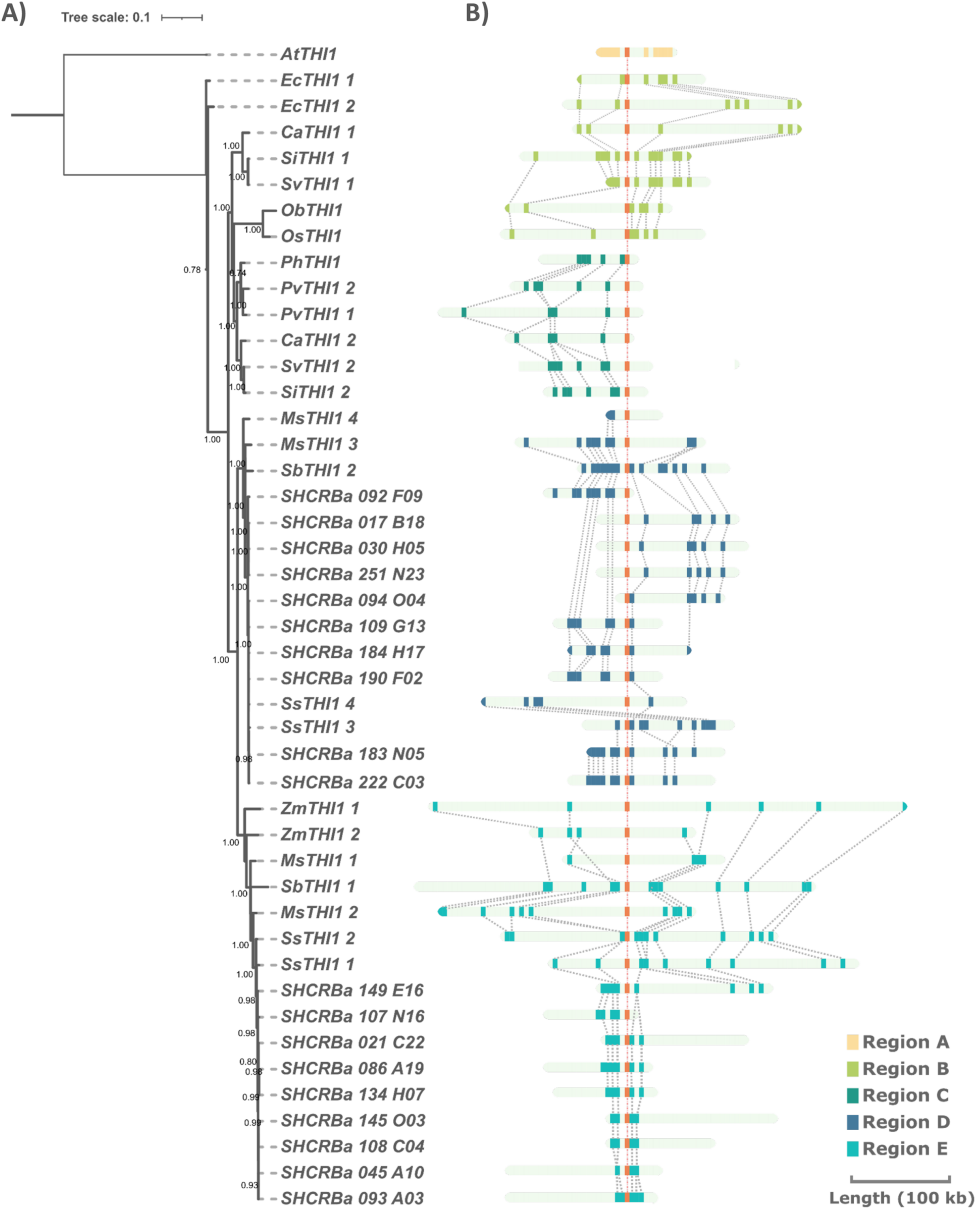
Comparison of *THI1* gene and its genomic flanking regions in sugarcane and other plants through phylogenetic and synteny analyses. (A) Numbers at nodes are branch support values estimated by the aLRT SHlike method implemented in PhyML3.0 (see Methods). The tree is based on the multiple alignments of the two exons of the single *THI1* copy from *A. thaliana* (*AtTHI1*), *E. coracana* (*EcTHI1*), *C. americanus* (*CaTHI1*), *O. sativa* (*OsTHI1*), *O. brachyantha* (*ObTHI1*), *S. italica* (*SiTHI1*), *S. viridis* (*SvTHI1*), *S. bicolor* (*SbTHI1*), *M. sinensis* (*MsTHI1*), *S. spontaneum* (*SsTHI1*) and BACs sequences of R570 sugarcane (BAC 108_C04, BAC 017_B18, and BAC 094_O04 represent *ScTHI1-1, ScTHI1-2a*, and *ScTHI1-2b*, respectively). (B) Synteny analysis was performed using blastx among sugarcane BAC sequences and sequences from the other genomes obtained from the plant’s database (see Table S2). Rectangles indicate genes.

To investigate the genomic context of *ScTHI1* homologs, we selected their genomic vicinities and compared them with TH1 phylogenetic relatedness. We identified five different genomic regions (A–E) flanking *THI1* genes (Fig. 2 and Table S3). *S. bicolor THI1* genes were found on chromosomes 2 (*SbTHI-2*) and 3 (*SbTHI1-2*) while in *Z. mays* the genes located on chromosomes 3 (*ZmTHI1-2*) and 8 (*ZmTHI1-1*). One copy of *THI1* has been identified in *O. brachyantha, O. sativa*, (both in chromosome 7) *and P. hallii* (chromosome 4). Homologs of *E. coracana* (chromosome 7), *C. americanus* (chromosomes 5 and 7), *S. italica and S. viridis* (both in chromosomes 2 and 4), and *P. virgatum* (chromosome 4) are also included in the present analysis.

Sugarcane *THI1* was found in two of these genomic loci (D and E) that by synteny are grouped in different *ScTHI1* clades. Two well-supported sugarcane clades (bootstrap 100 for *ScTHI1-1* and 99 for *ScTHI1-2*) were found and the clade of *ScTHI1-2* was divided into two subclades. BAC clones 017_B18, 030_H05, 251_N23, and 092_F09 cluster with *ScTHI1-2a* while BACs clones 094_O04, 109_G13, 183_N05, 184_N05, 190_F02, and 222_C03 with *ScTHI1-2b*, also with higher bootstrap values (Fig. 2).

### Molecular structure of THI1 homologues

**Figure 3:**
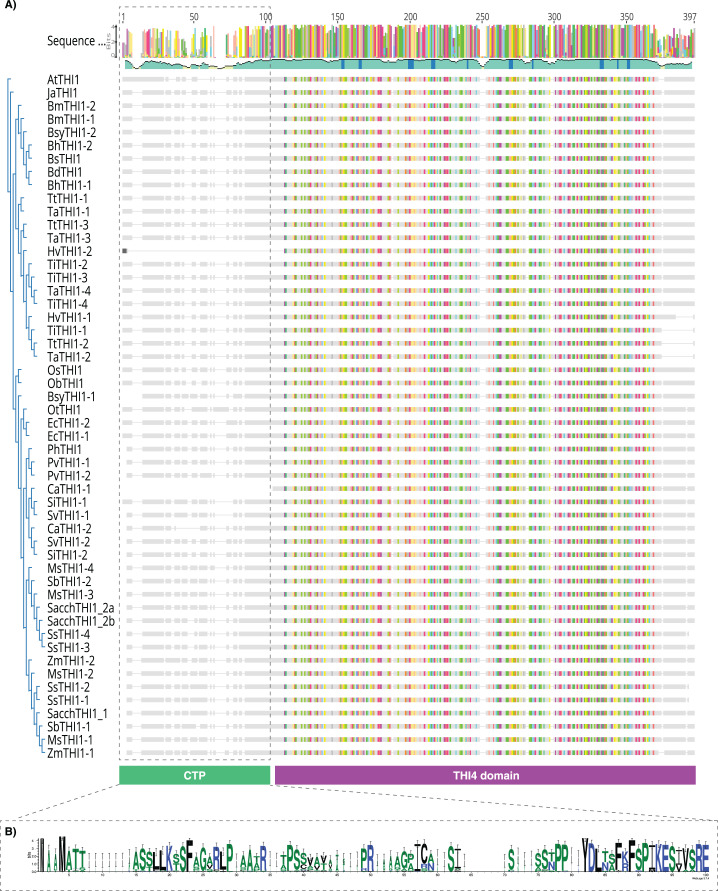
Alignment of amino acid sequences of THI1. (A) 51 THI1 sequences alignment across various species of Poaceae, using *J. ascendens* and *A. thaliana* as outgroups. The alignment was built with MAFFT v7. (B) WebLogo representation of multiple sequence alignment of N-terminal protein region indicating the relative frequency of amino acids at a given position (height). Abbreviation: CTP, Chloroplast Transient Peptide.

Exon-intron boundaries was examined for each of these 51 genes and the 19 BACs. While *AtTHI1* has two introns, all Poaceae species harbor only one intron with varying sizes (63 to 149 bp) (Fig. S1), suggesting high gene structure conservation. The protein length of the THI1 homologs is also highly conserved (Fig. 3A), with amino acid sequences at least 67% identical, including the less conserved region containing the previously described organellar targeting to mitochondria and/or chloroplast (Chabregas et al., 2001, 2003). Despite these features, the amino acid pre-sequence encoded by first start codon is variable, in length and amino acid content among species. Although the sequence and length of signal peptides can vary substantially, computational analyzes depict the existence of a series of conserved aminoacids at given positions, probably resulting in secondary structure conservation (see Fig. 3B). The signal peptide prediction results show that all *ScTHI1* proteins have a chloroplast targeting peptide when transcribed by the first initiation codon (1st ATG), as seen in other Poaceae species. The differences among the two sugarcane sequence subgroups lies at the 5′ region, where the Chloroplast Transit Peptide (CTP) is predicted (Machado et al., 1996).

Due to their high similarity, only one version of each ScTHI1 was taken into consideration for further detailed analysis of their nucleotide genomic sequence as described, ScTHI1-1 sequence was retrieved from BAC 108_C04 and ScTHI1-2a, ScTHI1-2b from BAC 017_B18, BAC 094_O04 respectively (Figs. S2A and S2B). When their CDS sequences were compared, high nucleotide identity and similarity were found (>91%) (Fig. 3B). Outside of the N-terminal region, only minor differences were detected mostly resulting in synonymous amino acid substitutions (Fig. S2C). The ScTHI1-1 protein differs from both ScTHI1-2 subgroups in one residue of MPS (Chabregas et al., 2003), and other seven residues along the protein (Fig. S2C).

### *THI1* promoter region analysis

**Figure 4:**
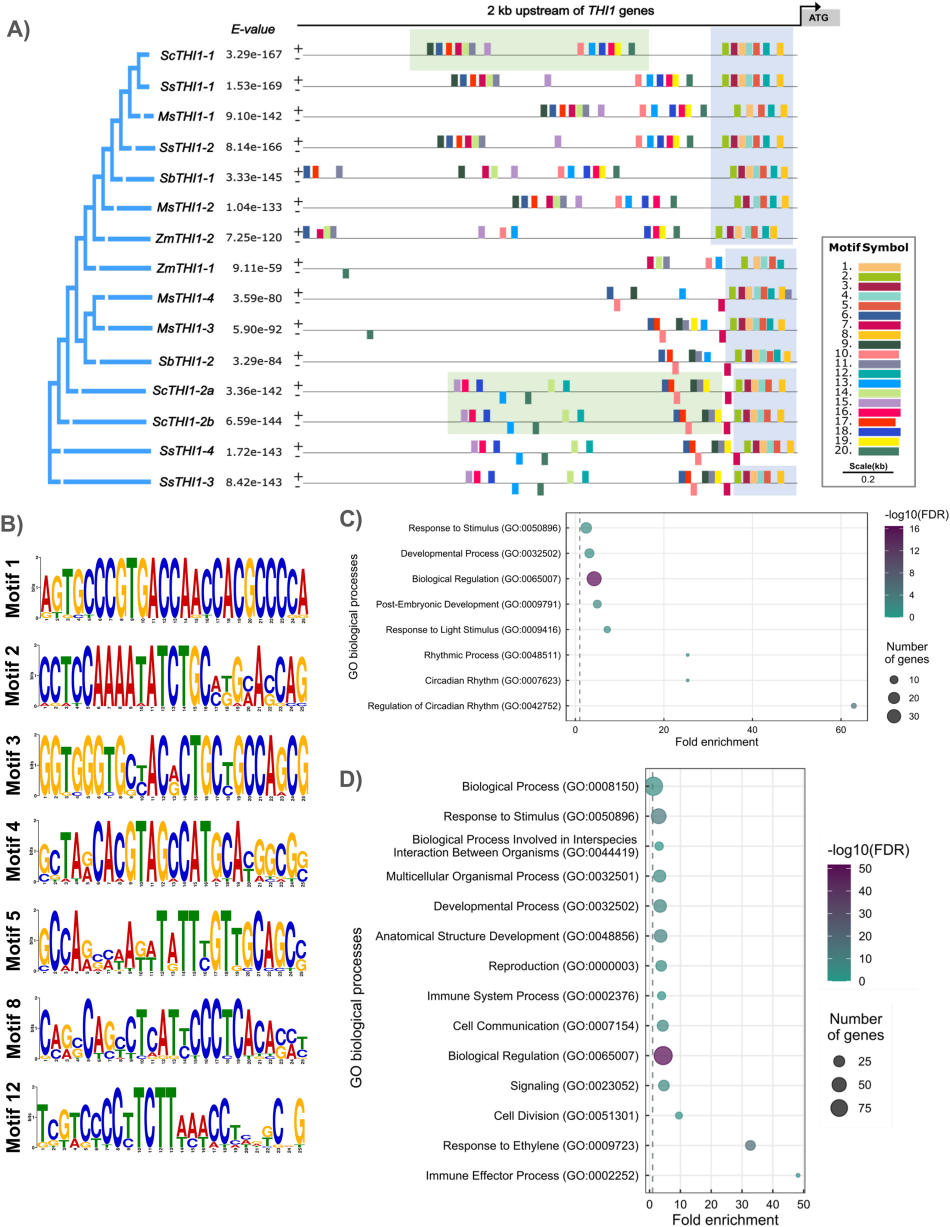
Promoter analysis and motif enrichment of *THI1* genes. (A) Motif identification in promoter regions (2,000 bp upstream of ATG) at *THI1* genes. The following parameters were used: site distribution = zero or one occurrence per sequence (zoops); minimum width = 5; minimum width = 25 bp; number of motifs = 20. The blue boxes showed conserved motifs among all grasses and the green boxes showed motifs present in *ScTHI1* paralogues. (B) Sequence logo of seven motifs (highly conserved) identified in the core promoter region of *THI1* of grasses. (C) GO enrichment analysis of motifs localized in the core promoter region of *THI1* of grasses (blue boxes in A). (D) GO enrichment analysis of motifs localized in the promoter region of *ScTHI1* (green boxes in A).

To understand the diversity of the *ScTHI1* promoter region in sugarcane, we performed an *in-silico* analysis of the 2 kb sequence preceding the start codon of the predicted genes (region D and E, Fig. 2). First, we identified 20 conserved motifs with sizes between 5 and 25 bp among the five species with the highest genome conservation (*i.e*., *M. sinensis*, *S. bicolor*, sugarcane, *S. spontaneum*, and *Z. mays*). Our analyses revealed different degrees of conservation between the motifs (support statistical of *p*-value ≤ 0.05), in which a group of seven motifs formed a standard portion among the species (Fig. 4A). The promoter regions of these genes were highly conserved on the 300 bp next to the start codon, where at least seven motifs were common among 15 analyzed sequences. Therefore, this region was considered as the core promoter (Fig. 4B). The promoter regions of the three variants of the sugarcane var. R570, *ScTHI1-1, ScTHI1-2a* and *ScTHI1-2b*, shared most *cis*-regulatory elements (CREs). However, ScTHI1-1 presented a unique distribution and organization, while *ScTHI1-2a* and *ScTHI1-2b* shared as many CREs as distribution, supporting the presence of ScTHI1 in sugarcane along two genomic regions (Fig. 4A).

Next, transcription factors (TFs) with the potential to bind to CREs were identified in all 20 motifs (*p*-value ≤ 0.01), and GO terms association allowed us to understand the biological processes in which they are involved (see Fig. 4C). The GO terms of the core promoter (highlighted in blue in Fig. 4A) revealed enrichment on biological processes related to: ‘Biological regulation’ (GO:0065007), ‘Developmental process’ (GO:0032502), ‘Post-Embryogenic development’ (GO:0009791), ‘Response to a stimulus’ (GO:0050896), ‘Response to a light stimulus’ (GO:0009416), ‘Rhythmic process’ (GO:0048511), ‘Circadian rhythm’ (GO:0007623), and ‘Regulation of circadian rhythm’ (GO:0042752). Fitzpatrick & Noordally (2021) reported the relationship between the genes of the thiamine pathway and the influence of light/dark transitions, suggesting a daily transcription regulation of genes involved in the synthesis of vitamin B1.

When considering only the genes from sugarcane, TFs with potential binding in the CREs of the *ScTHI1-1* and *ScTHI1-2* promoters (highlighted in green in Fig. 4A), show functional redundancy because both GO terms are related to ‘Response to stimulus’ (GO:0050896), ‘Developmental process’ (GO:0032502), and ‘Biological regulation’ (GO:0065007). These results reinforce the putative biological identity of THI1 regulation in the thiamine pathway and its role in coordinating plant development processes. Additionally, the enrichment of terms related to plant-microorganism interaction was observed, such as ‘Biological process involved in interspecies interaction between organisms’ (GO:0044419), ‘Immune system process’ (GO:0002376), and ‘Immune effector process’ (GO:0002252), in as well as GO terms associated with ‘Signaling’ (GO:0023052) and ‘Cell communication’ (GO:0007154). Although these *in silico* observations still need to be confirmed experimentally, studies in *A. thaliana*, rice, and other crops (tobacco, cucumber, and tomato) have already demonstrated the participation of thiamine during the process of infection by pathogenic microorganisms (Ahn, Kim & Lee, 2005) and response to oxidative stress (Tunc-Ozdemir et al., 2009), Moreover, improvement in infection resistance was described for mutants of *A. thaliana* and *O. sativa* (Dong, Stockwell & Goyer, 2015; Dong et al., 2016).

### *THI1* sequence diversity in modern sugarcane cultivars

In order to address the haplotype sequence diversity of *THI1* in the *Saccharum* complex, we designed a primer pair in a conserved region between amino acids 49 to 161 (Fig. S2C). This conserved region was amplified from the closely related species *Miscanthus sp, S. officinarum*, *S. spontaneum* and 10 modern sugarcane hybrids cultivars. Table S4 describe how many sequences of the copies of *ScTHI1* were amplified for each of the genotypes/varieties.

**Figure 5:**
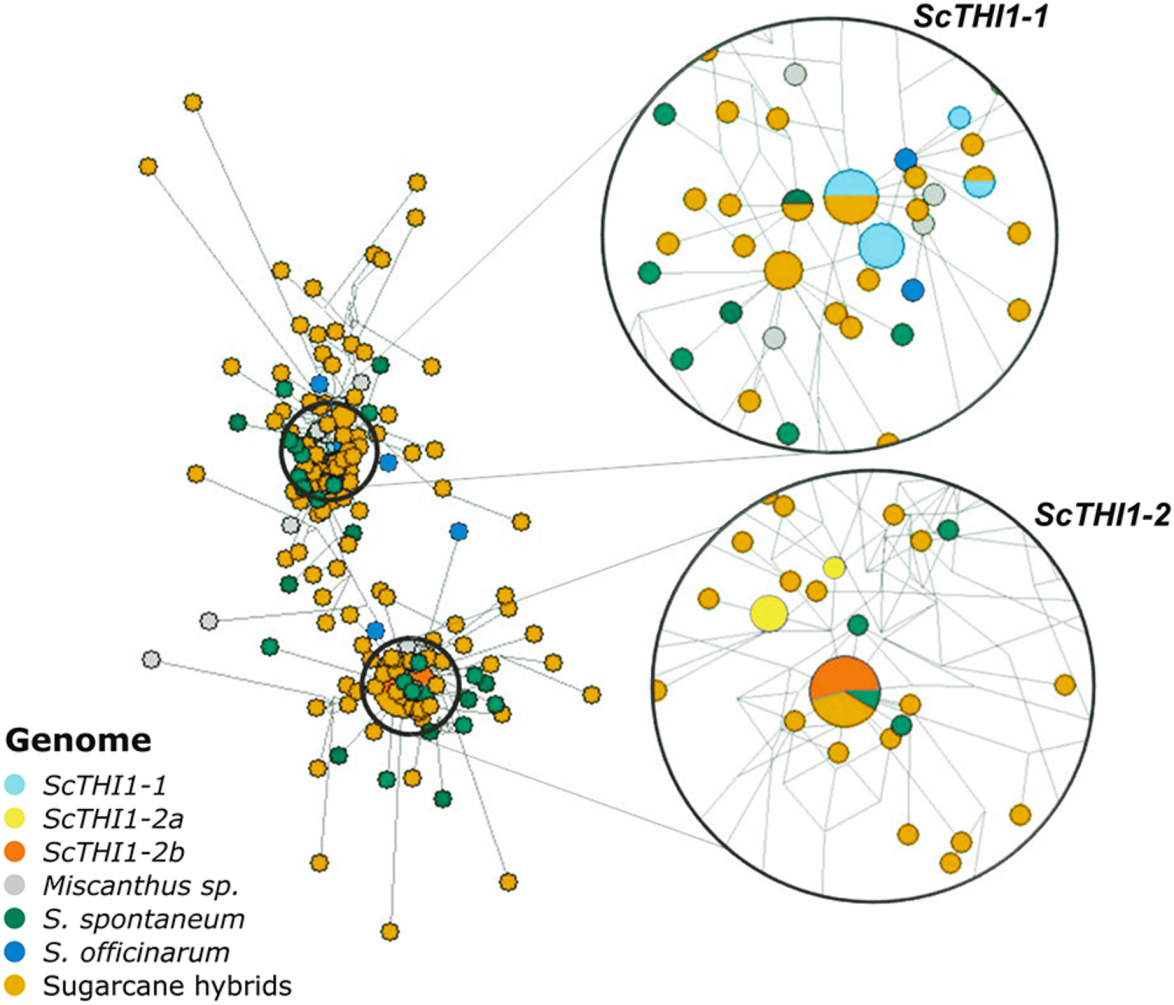
Network analysis of *THI1* gene in *Saccharum* complex. The network was built using the NETWORK 4.6.1.3 software (Bandelt, Forster & Röhl, 1999) with default parameters (Median-joining method). An alignment of a region of 539 bp of 210 sequences of varieties of sugarcane was used to construct the network. The right part of the figure is a close-up of the entire network shown on the left. The size of the circles is proportional to the number of sequences in the haplotype; the distance between clusters is proportional to the number of substitutions observed between sequences.

Amplicons varying between 321–330 bp each were sub-cloned and 197 were sequenced, assembled and aligned against the 19 BAC sequences. A network analysis was performed (Fig. 5), where two main clusters emerge that are not species- or cultivar-specific, but *ScTHI1* copy-specific (Table S4) (Vieira, 2018).

### Developmental and tissue-specific expression profiles of sugarcane *ScTHI1* genes

**Figure 6:**
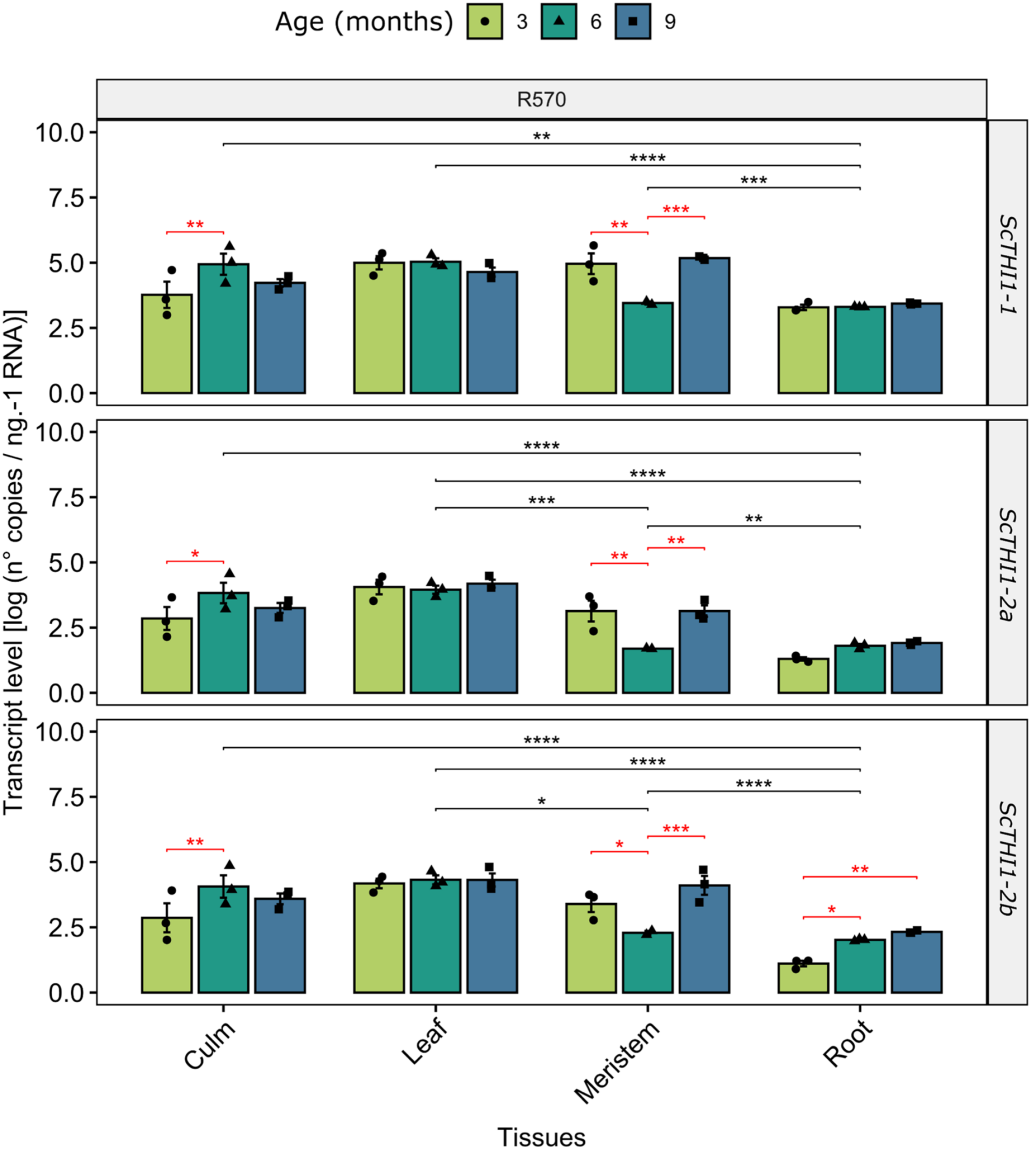
Expression analysis of *ScTHI1* genes in different tissues and ages in the sugarcane cultivar R570. Data are the means of three biological replicates; error bars indicate SD. Asterisks indicate significant differences among compared groups using the ANOVA and *p*-adjust (Bonferroni method) <0.05 (*), 0.01(**), 0.001 (***), and 0.0001(****). Black and red lines represent statistically significant differences between tissues and ages.

To gain insights into how *ScTHI1* genes are expressed in sugarcane we investigated changes in their expression patterns in different tissues (meristem, root, culm, and leaf) along the development of *Saccharum sp*. var R570. Our analysis revealed that all identified variants were expressed in all tissues and ages (Fig. 6). Furthermore, differential expression was detected when comparing different tissue (ANOVA *p*-value ≤ 0.01) and age, the latter more significantly evidenced in the meristematic tissue for all *ScTHI1* variants (Fig. 6). In culm, a change in expression level was seen in the transition from 3 to 6 months, corroborating previous findings (Partida et al., 2021).

### Functional complementation assay

A *Saccharomyces cerevisiae* mutant strain, KBY5 (THI4::URA3), has a truncated *THI4* gene, which impairs growth on a minimal medium without thiamine supplementation. Machado et al. (1996) reported that *A. thaliana* homolog (*AtTHI1*) can restore the thiamine auxotrophy of this strain. An analogous complementation assay using *ScTHI1-1*, *ScTHI1-2a*, and *ScTHI1-2b* was performed. Two versions of each of the three CDSs were synthesized and transferred to the yeast expression vector pG-1 (Vieira, 2018). One version represents the full *ScTHI1-1*, *ScTHI1-2a*, and *ScTHI1-2b* CDS and the second version is the CDS lacking the N-terminal chloroplast transit peptide sequences, named *ScTHI1-1/DelN*, *ScTHI1-2a/DelN*, and *ScTHI1-2b*/DelN. Two other constructs, A184V and DelN in the same expression vector were used as controls. A184V is the cDNA of the *THI1* gene from the *A. thaliana tz-201* mutant and was used as positive control. In fact, it served as a control for partial complementation (Papini-Terzi et al., 2003). The negative control DelN contains a cDNA of the wild-type *AtTHI1* gene without the N-terminal chloroplast transit peptide (Papini-Terzi et al., 2003). Growth was evaluated after 4 and 28 days of incubation at 30 °C.

**Figure 7:**
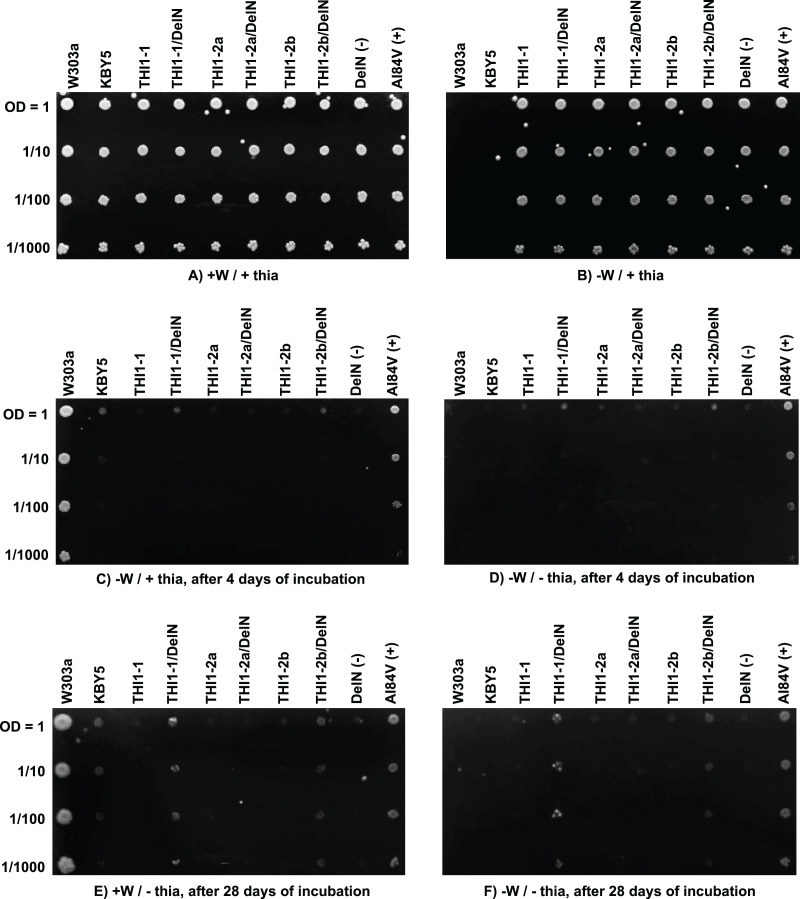
Functional yeast complementation assay. *S. cerevisiae* strain KBY5 was transformed with the three versions of the *ScTHI1* gene found in the sugarcane genome, with the positive control *A184V* and the negative control and DelN, and plated in YNB media with or without tryptophan and thiamine. W303a strain was used as positive control. W = tryptophan and “thia” = thiamine. Each column represents one transformant. Lines represent dilution series. (A and B) are the experimental controls to check if all strains can grow (A) and if all transformed ones can grow without tryptophan (B). (C and D) show growth after 4 days of incubation, while (E and F) after 28 days of incubation, at 30 °C.

KBY5 strain transformed independently with the three sugarcane constructs and plated on YNB medium supplemented with thiamine (thia) and tryptophan (W) could efficiently grow (Fig. 7A). The inability of the wild-type strain W303a and KBY5 to grow on selective [−W/+ thia] YNB medium shows that the transformation with the sugarcane constructs was efficient and complemented W auxotrophy in the recipient strain (Fig. 7B). Only wild type strain W303a and auxotrophic complemented strains by the recipient plasmid could grow on plates supplemented with W only after four (Fig. 7C) and 28 (Fig. 7E) days of incubation. None of the full-length *ScTHI1* CDSs complemented KBY5 thiamine auxotrophy, but both DelN versions of *ScTHI1-1* and *ScTHI1-2b* did. However, this complementation was less efficient than the *A. thaliana* A184V allele (Papini-Terzi et al., 2003), with yeasts forming smaller colonies and taking more time to develop.

## Discussion

Studying precursor genes for coenzymes has been of increasing interest in biology, especially in plant crops (Pourcel, Moulin & Fitzpatrick, 2013; Strobbe et al., 2021). For many years, plant science has focused on genes directly responsible for the increase in biomass. More recently, several studies have looked at coenzymes and their impact on metabolic pathways, especially on the carbohydrate (see review of Fitzpatrick & Noordally (2021)). Here, we show an integrative view for understanding the biology of the *THI1* genes Poaceae, especially in sugarcane. We aimed at elucidating this gene’s structure, organization, and distribution in the genomes and clarifying aspects of the regulation of *THI1* expression, targeting, and gene diversity.

Sugarcane is an important tropical crop, cultivated for the production of sucrose and bioethanol. Several efforts for sequencing its complex genome structure have been made and the most complete managed to cover 373,869 genes (99.1% of the sugarcane genome) (Souza et al., 2019). The unavailability of the full genome poses a challenge in investigating the genomic context of genes in this species. However, our study provides the first complete overview of *THI1* in Poaceae, including protein characterization, phylogeny, gene structure, chromosome location, synteny, and gene expression patterns.

### Features of *THI1* genes have been conserved during Poaceae speciation

Our genome-wide investigation of *THI1* genes include all Poaceae species sequenced to date, which have at least one *THI1* homologue gene. The aligned sequences displayed that with the exception of the variable N-terminal region, most of the nucleotide changes have resulted in synonymous substitution. The variability found at the N-terminal region could be explained by its targeting function that depends on the classes of amino acids (hydrophobic and positively charged) present rather than on specific amino acid sequences (von Heijne & Gavel, 1988; Käll, Krogh & Sonnhammer, 2004). As a result, this region could accommodate non-synonymous substitutions without loss of function.

In addition to the subgroups formed among ScTHI1, the homologous of the species *S. italica, S. viridis, C. americanus, P. virgatum, E. coracana* and *P. hallii* form a group (Paniceae) sister to the Andropogoneae tribe. Interestingly, members of Bambusoideae, Oryzoideae, and Pooideae subfamilies (B.O.P) grouped together in the clade comprised by *B. distachyon*, *B. hybridum, B. mexicanum, B. stacei*, *B. sylvaticum*, *T. intermedium, T. aestivum, and T. turgidum*. Despite the species *O. sativa* and *O. brachyantha* being part of this same clade (B.O.P), it is possible to see that the homologs of the genus *Oryza* are less related. The topology of the evolutionary tree of the predicted amino acid sequences from THI1 of Poaceae was similar to the one described by Soreng et al. (2015, 2017). Furthermore, homologs of the same species clustered together, indicating that these duplication events happened independently after speciation.

Despite the variation found in the N-terminal region, THI4 domains are reported to be highly conserved (Hwang et al., 2014). The exceptions were THI1 homologs non-Cys present in *T. aestivum, T. intermedium, T. turgidum*, and *H. vulgare*. The expression levels of non-Cys variants were found to be lower than those with this cysteine residue conserved (Joshi et al., 2020). Similar to Archaea species, such as *Methanococcus igneus* (Zhang et al., 2016) and *Methanocaldococcus jannashi* (Eser et al., 2016), the obtention of thiazole ring is accomplished by the use of the nicotinamide adenine dinucleotide, glycine and free sulfide. This suggests that a THI1 isoform non-Cys is only restricted to those cereal species, which possibly need a Cys containing THI1 isoform capable of donating a sulfur molecule to form the thiazole ring.

THI1 has a central role in thiamine biosynthesis that in turn is an essential cofactor for several metabolic pathways, such as amino acids metabolism (Duggleby, 2006; Duggleby, McCourt & Guddat, 2008) and carbohydrate (Belanger et al., 1995). According to the amino acid sequences analyzed, the residue (Cys205) required for the Fe^2+^-binding (Zhang et al., 2016; Eser et al., 2016; Joshi et al., 2020) is fully conserved in THI1 from Poaceae. This Cys residue is known to be the sulfur donor in yeast (Chatterjee et al., 2011), plants (Godoi et al., 2006), and the Archaea species *H. volcanii* (Hwang et al., 2014). Our results indicate that THI1 sulfur donation function is present at least in one gene copy and is suggestive of its role in the thiazole ring formation in all Poaceae isoforms.

The molecular characterization of the *THI1* gene revealed that gene duplication has not only occurred in C4 plants and that *THI1* genes are also positioned in different genomic regions in Poaceae, probably due to the number of genome duplications in those groups (de Setta et al., 2012; Svačina et al., 2020; Lee et al., 2020). Recent studies based on comparative genomics support the occurrence of whole-genome duplication in angiosperms (Jiao et al., 2011) and early polyploidy in monocots (Tang et al., 2010). Furthermore, our study shows that C3 species generally have a single copy of *THI1*. However, this is not the case for wheat and barley, representatives of the Triticeae group that are the most prominent example of duplication under the influence of the domestication event (Qiao et al., 2019).

The comparative analysis identified five non-syntenic genomic regions among the Poaceae genomes. Evidence of the *THI1* gene duplications and translocations as well as duplications of genomic regions containing this gene are presented. The common ancestor of the Andropogoneae tribe (sugarcane, sorghum, and maize) has two loci carrying *THI1* copies (*THI1-1*—present in region D and *THI1-2*—present in region E Fig. 2), which is observed in high collinearity between sugarcane and sorghum chromosomes (Ming et al., 1998; Vieira, 2018). *S. italica* and *S. viridis* have two *THI1* copies, one in a region syntenic to those of *C. americanus, O. sativa, O. brachyantha*, and *E. coracana* (region B). Another copy is located in a different area shared among *P. hallii, P. virgatum* and other copy of the *C. americanus* (region C). *Z. mays* duplicated the entire region E. Finally, the *A. thaliana THI1* region is non-syntenic to grasses genomic regions analyzed here (region A). These results provide an overview of Poaceae *THI1*, including their gene numbers, evolutionary relationship, and structural conservation locus.

### *ScTHI1* genes share conserved molecular features

We identified 19 BACs of sugarcane var. R570 containing *THI1-*like genes. Our genomic characterization revealed at least two groups of *ScTHI1* genes, nine alleles of *ScTHI1-1* and ten alleles of *ScTHI1-2* (Vieira, 2018). Phylogenetic (Fig. 1B) and network analyses (Fig. 5) of its sequences showed that, despite the similarity among *ScTHI1-2* genes, a diversification of *ScTHI1-2* has occurred. Two subgroups were identified, including four alleles of the *ScTHI1-2a* group and six alleles of the *ScTHI1-2b* group.

As shown in the synteny analysis, each paralogue of *ScTHI1* is present in a different genomic environment. In addition, the core promoter located 600 bp upstream the start codon (ATG) is highly conserved, among the sugarcane ScTHI1 paralogs and Poaceae. Further analysis of the promoter regions (2 kb upstream from ATG) revealed that *ScTHI1.1* and *ScTHI1.2* have distinct sets of of CRE and TF binding sites conserved across all *Saccharum* species, which contributed to the prediction of their potential function. Together, these data support the idea of a gene duplication occurring in a common ancestor of the Andropogoneae tribe, preserving the gene and its core promoter region along the evolution.

The diversification of two *ScTHI1-2* subgroups is supported by the Network analysis. *ScTHI1-2b* sequences fall into one haplotype along with one *S. spontaneum* and four modern cultivars sequences, whereas *ScTHI1-2a* sequences fall into two distinct haplotypes, one comprised of several BACs (017_B18, 030_H05 and 251_N23) and a second composed by 092_F09.

Our data revealed that the expression pattern between the variants is very similar. Looking only at the leaf tissue, where the three variants showed constantly higher expression levels over the development, a 10-fold difference in expression of *ScTHI1-1* compared to *ScTHI1-2a* and 5-fold compared to *ScTHI1-2b* is seen. Comparing the different tissues, the leaf presents the largest number of transcripts while the root has the lowest number, showing the relationship of *ScTHI1* with photosynthetic tissues, as previously reported for *Alnus glutinosa* (Ribeiro et al., 1996), Arabidopsis (Papini-Terzi et al., 2003; Ribeiro et al., 2005), and the crops species cassava (Mangel et al., 2017), wheat (Joshi et al., 2020), and barley (Joshi et al., 2020). In addition, our results of CREs in the promoter region of *ScTHI1* reveals potential binding sites to TFs related to development process corroborating the expression data.

Our complementation assays revealed that the three full-length copies of *ScTHI1* could not complement the KBY5 strain. Papini-Terzi et al. (2003) also presented that this strain grows poorly after 4 days of cultivation when bearing the A184V construction. The ScTHI1-1 DelN and ScTHI1-2b DelN transformants were partially complemented but took longer (28 days) to develop, suggesting that the chloroplast transit peptide at the N-terminus of THI1 from sugarcane somehow interferes with the complementation efficiency in the KBY5 yeast strain. *ZmTHI1-1* and *ZmTHI1-2* can restore thiamine prototrophy in yeast (Belanger et al., 1995).

## Conclusions

Taken together, the study of the sugarcane *THI1* supports the existence of multiple independent rounds of gene duplication events involving *THI1* orthologs. Each tribe presents its unique genomic *THI1* environment except maize which shares the same environment for the two gene copies. Expression of sugarcane *THI1* is redundant across tissues and developmental stages where the leaf presents the higher expression level and the root the least. This is consistent with the similarity observed at the core promoter of the paralog genes; however, subtle intensity changes demand dissecting the expression differences in more detail. Sugarcane gene copies are redundant at the transcription level, and two of the three copies are functionally redundant. Further studies are needed to explore the contribution of the levels of the thiazole ring in C4 photosynthetic plant tissues or potentially the relevance of the THI1 protein activity.

### Accession numbers

Sequence data from this article can be found in NCBI. Accession numbers: KF184731.1; KF184959.1; KF184877.1; KF184851.1; KF184849.1; KF184930.1; KF184747.1; KF184858.1; KF184724.1; PRJEB26915

## Supplemental Information

10.7717/peerj.14973/supp-1 Supplemental Information 1 Gene structure of thiamine thiazol synthase (*THI1*) in Poaceae. Exon/intron organization of *THI1* genes was depicted with the online Gene Structure Display Server (GSDS). The exons and introns are represented by green boxes and black lines, respectively, and the blue boxes represent the 5′ and 3′ UTR.

10.7717/peerj.14973/supp-2 Supplemental Information 2 Sequence characterization of *ScTHI1* genes in BACs of sugarcane (*Saccharum sp*. var. R570). (A) Multiple sequence alignment of the *ScTHI1* CDS identified on BACs library using the program MAFFT v7. (B) Percentage of identity among the three *ScTHI1* CDS. (C) Multiple alignments of amino acid sequences of the ScTHI1 selected to represent each group. The dotted line region was used in the Network analysis (see Methods). Color shades represent the identities and similarities among amino acids, with a threshold of 100%. The red arrow points to Cys residue depicted in Figure C.

10.7717/peerj.14973/supp-3 Supplemental Information 3 Selected BACs with *ScTHI1* presence. Features of BACs and *ScTHI1* CDS size by SAS probe.

10.7717/peerj.14973/supp-4 Supplemental Information 4 *THI1* genes identified in Poaceae genomes. Gene IDs, genomic location, and protein features of Poaceae *THI1* genes. The gene IDs given were obtained from Phytozome 13 (https://phytozome-next.jgi.doe.gov/) and PLAZA Monocots v4.5 (https://bioinformatics.psb.ugent.be/plaza/versions/plaza_v4_5_monocots/).

10.7717/peerj.14973/supp-5 Supplemental Information 5 Accession numbers of the sequences used in synteny analysis by species.

10.7717/peerj.14973/supp-6 Supplemental Information 6 Clones assembled from each genome. Total number and number related to each *ScTHI1* copy.

## Additional Information and Declarations

### Data Availability

The following information was supplied regarding data availability: The raw data is available in the Supplemental Tables and at GenBank: KF184731.1, KF184959.1, KF184877.1, KF184851.1, KF184849.1, KF184930.1, KF184747.1, KF184858.1, KF184724.1, PRJEB26915.
