# Supplementary figures and images for: Functional and comparative analysis of THI1 gene in grasses with a focus on sugarcane
Source: PeerJ. 2023 May 15;11:e14973. doi: 10.7717/peerj.14973 (PMC10194071; doi:10.7717/peerj.14973)

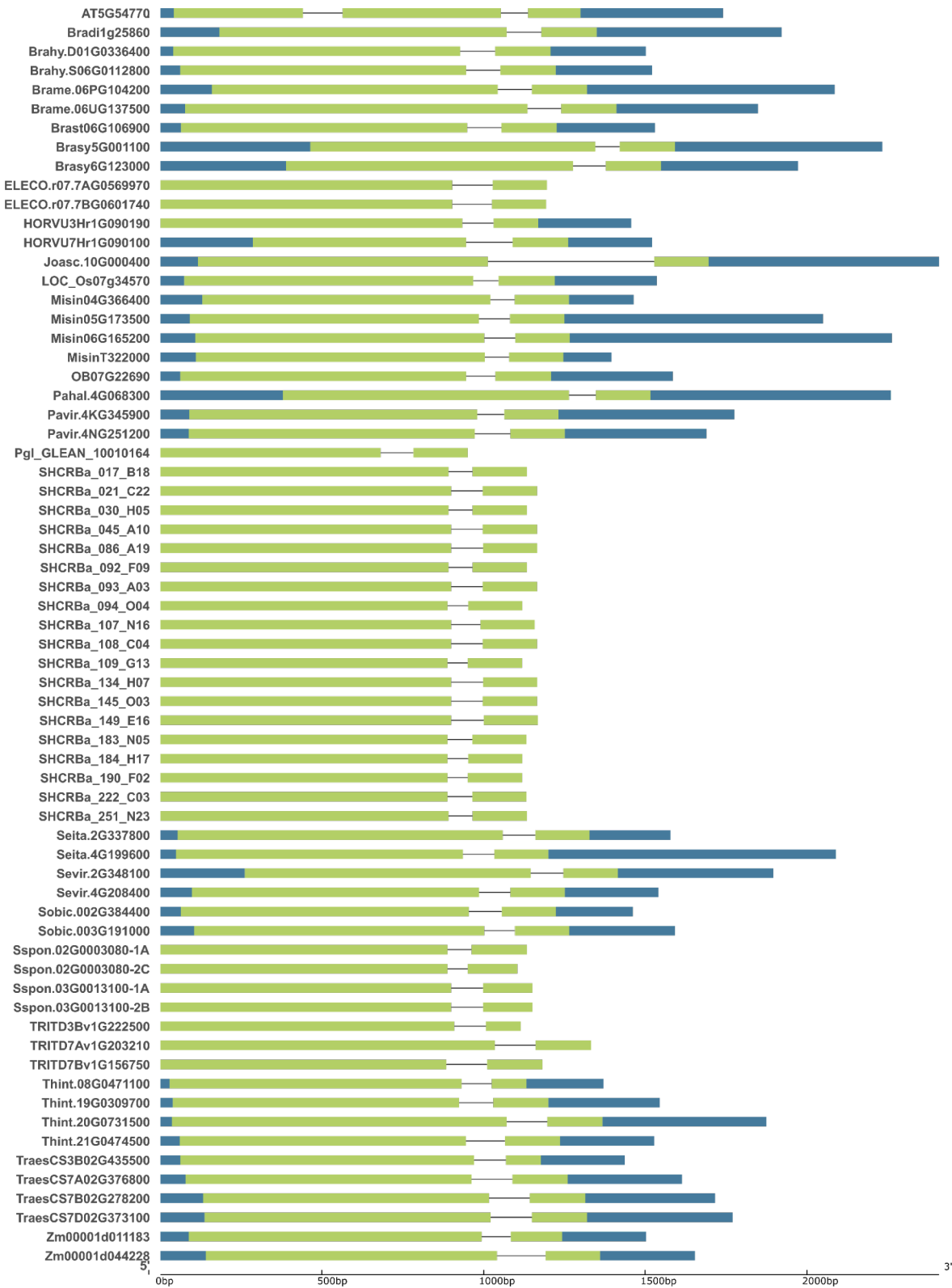

Legend:

CDS upstream/ downstream Intron

Supplement: Supplemental Information 1 — Exon/intron organization of THI1 genes was depicted with the online Gene Structure Display Server (GSDS). The exons and introns are represented by green boxes and black lines, respectively, and the blue boxes represent the 5′ and 3′ UTR. [file peerj-11-14973-s001.pdf]
