# Supplementary material for: Functional and comparative analysis of THI1 gene in grasses with a focus on sugarcane
Source: PeerJ. 2023 May 15;11:e14973. doi: 10.7717/peerj.14973 (PMC10194071; doi:10.7717/peerj.14973)

A)

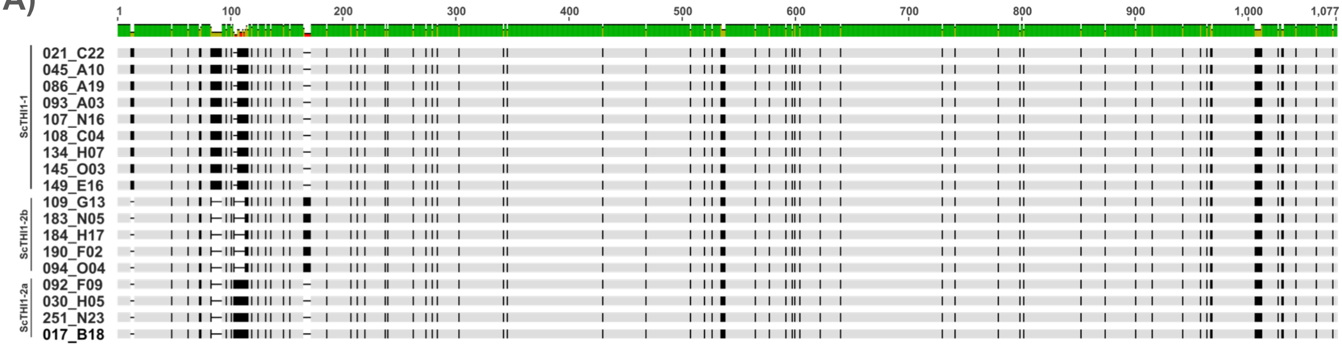

B)

ScTHI1-1	**100%**		
ScTHI1-2a	**93.23%**	**100%**	
ScTHI1-2b	**91.34%**	**98.22%**	**100%**
CDS	***ScTHI1-1***	***ScTHI1-2a***	***ScTHI1-2b***

C)

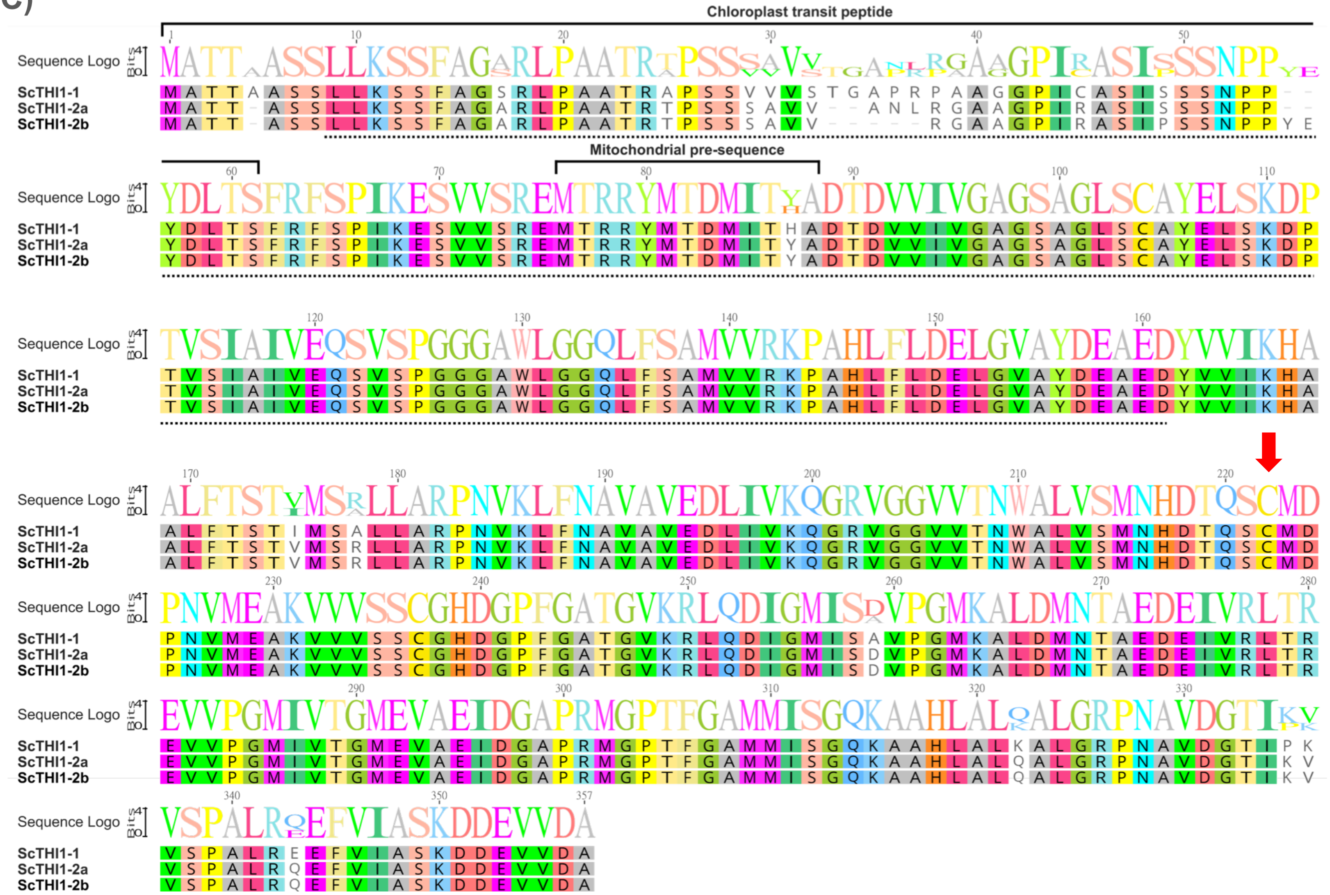

Supplement: Supplemental Information 2 — (A) Multiple sequence alignment of the ScTHI1 CDS identified on BACs library using the program MAFFT v7. (B) Percentage of identity among the three ScTHI1 CDS. (C) Multiple alignments of amino acid sequences of the ScTHI1 selected to represent each group. The dotted line region was used in the Network analysis (see Methods). Color shades represent the identities and similarities among amino acids, with a threshold of 100%. The red arrow points to Cys residue depicted in Figure C. [file peerj-11-14973-s002.pdf]
