# Supplementary material for: Functional and comparative analysis of THI1 gene in grasses with a focus on sugarcane
Source: PeerJ. 2023 May 15;11:e14973. doi: 10.7717/peerj.14973 (PMC10194071; doi:10.7717/peerj.14973)
Supplement: Supplemental Information 3 — Features of BACs and ScTHI1 CDS size by SAS probe. [file peerj-11-14973-s003.docx]

**Table S1. Selected BACs with *ScTHI1* presence.** Features of BACs and *ScTHI1* CDS size by SAS probe.

| **Probe** | **SHCRBa BAC name** | **BAC size (bp)** | ***ScTHI1* CDS size (bp)** |
| --- | --- | --- | --- |
| **SAS1_*ScTHI1*** | 021_C22 | 135,265 | 1,068 |
|  | 045_A10 | 145,524 | 1,068 |
|  | 086_A19 | 101,298 | 1,068 |
|  | 093_A03 | 139,698 | 1,068 |
|  | 107_N16 | 87,694 | 1,068 |
|  | 108_C04 | 101,728 | 1,068 |
|  | 134_H07 | 97,816 | 1,068 |
|  | 145_O03 | 157,314 | 1,068 |
|  | 149_E16 | 164,894 | 1,068 |
| **SAS2_*ScTHI1*** | 017_B18 | 131,240 | 1,059 |
|  | 030_H05 | 141,404 | 1,059 |
|  | 092_F09 | 85,055 | 1,059 |
|  | 251_N23 | 130,963 | 1,059 |
|  | 094_O04 | 102,271 | 1,056 |
|  | 109_G13 | 100,204 | 1,056 |
|  | 183_N05 | 129,178 | 1,056 |
|  | 184_H17 | 115,565 | 1,056 |
|  | 190_F02 | 105,074 | 1,056 |
|  | 222_C03 | 138,508 | 1,056 |
