# Supplementary material for: Functional and comparative analysis of THI1 gene in grasses with a focus on sugarcane
Source: PeerJ. 2023 May 15;11:e14973. doi: 10.7717/peerj.14973 (PMC10194071; doi:10.7717/peerj.14973)
Supplement: Supplemental Information 4 — Gene IDs, genomic location, and protein features of Poaceae THI1 genes. The gene IDs given were obtained from Phytozome 13 (https://phytozome-next.jgi.doe.gov/) and PLAZA Monocots v4.5 (https://bioinformatics.psb.ugent.be/plaza/versions/plaza_v4_5_monocots/). [file peerj-11-14973-s004.docx]

**Table S2. *THI1* genes identified in Poaceae genomes.** Gene IDs, genomic location, and protein features of Poaceae *THI1* genes. The gene IDs given were obtained from Phytozome 13 (https://phytozome-next.jgi.doe.gov/) and PLAZA Monocots v4.5 (https://bioinformatics.psb.ugent.be/plaza/versions/plaza_v4_5_monocots/).

| **Photosynthetic system** | **Organism** | **Gene name** | **ID Database** | **Database** | **Chromossome number / Chromossome position** | **Strand** | **CDS lenght (bp)** | **Genomic sequence lenght (bp)** | **Protein lenght (aa)** |
| --- | --- | --- | --- | --- | --- | --- | --- | --- | --- |
| C3 | *Arabidopsis thaliana* | AtTHI1 | *AT5G54770* | Phytozome 13 | Chr05:22246591-22248332 | + | 1050 | 1741 | 350 |
| C3 | *Brachypodium distachyum* | *BdTHI1* | *Bradi1g25860* | Phytozome 13 | Chr01:20986491-20988413 | - | 1059 | 1922 | 353 |
| C3 | *Brachypodium hybridum* | *BhTHI1-1* | *Brahy.D01G0336400* | Phytozome 13 | Chr01D:20735968-20737470 | - | 1059 | 1502 | 353 |
| C3 | *Brachypodium hybridum* | *BhTHI1-2* | *Brahy.S06G0112800* | Phytozome 13 | Chr06S:6806970-6808491 | - | 1056 | 1521 | 352 |
| C3 | *Brachypodium mexicanum* | *BmTHI1-1* | *Brame.06PG104200* | Phytozome 13 | Chr06P:16798042-16800128 | - | 1056 | 2086 | 352 |
| C3 | *Brachypodium mexicanum* | *BmTHI1-2* | *Brame.06UG137500* | Phytozome 13 | Chr06U:24737184-24739033 | - | 1230 | 2086 | 410 |
| C3 | *Brachypodium stacei* | *BsTHI1* | *Brast06G106900* | Phytozome 13 | Chr06:6793052-6794582 | - | 1056 | 1530 | 352 |
| C3 | *Brachypodium sylvaticum* | *BsyTHI1-1* | *Brasy5G001100* | Phytozome 13 | Chr05:164231-166465 | + | 1059 | 1973 | 353 |
| C3 | *Brachypodium sylvaticum* | *BsyTHI1-2* | *Brasy6G123000* | Phytozome 13 | Chr06:9164176-9166149 | - | 1053 | 2234 | 351 |
| C3 | *Hordeum vulgare* | *HvTHI1-1* | *HORVU3Hr1G090190* | PLAZA Monocot 4.5 | Chr03H:630892988-630894155 | - | 1081 | 1471 | 359 |
| C3 | *Hordeum vulgare* | *HvTHI1-2* | *HORVU7Hr1G090100* | PLAZA Monocot 4.5 | Chr07H:547824267-547825250 | - | 1116 | 1873 | 372 |
| C3 | *Joinvillea ascendens* | *JaTHI1* | *Joasc.10G000400* | Phytozome 13 | Chr10:52849-55258 | + | 1065 | 2409 | 355 |
| C3 | *Oryza brachyantha* | *ObTHI1* | *OB07G22690* | PLAZA Monocot 4.5 | Chr07:11657958-11659104 | + | 1056 | 2785 | 351 |
| C3 | *Oryza sativa* | *OsTHI1* | *LOC_Os07g34570* | Phytozome 13 | Chr07:20724562-.20726098 | + | 1068 | 1536 | 356 |
| C3 | *Thinopyrum intermedium* | *TiTHI1-1* | *Thint.08G0471100* | Phytozome 13 | Chr08:314323786-314325157 | - | 1011 | 1371 | 337 |
| C3 | *Thinopyrum intermedium* | *TiTHI1-2* | *Thint.19G0309700* | Phytozome 13 | Chr19:218529608-218531153 | - | 1056 | 1545 | 352 |
| C3 | *Thinopyrum intermedium* | *TiTHI1-3* | *Thint.20G0731500* | Phytozome 13 | Chr20:492858362-492860237 | + | 1206 | 1875 | 402 |
| C3 | *Thinopyrum intermedium* | *TiTHI1-4* | *Thint.21G0474500* | Phytozome 13 | Chr21:313971607-313973135 | + | 1056 | 1528 | 352 |
| C3 | *Triticum aestivum* | *TaTHI1-1* | *TraesCS7A02G376800* | PLAZA Monocot 4.5 | Chr07A:550052099-550053280 | + | 1066 | 1630 | 354 |
| C3 | *Triticum aestivum* | *TaTHI1-2* | *TraesCS3B02G435500* | PLAZA Monocot 4.5 | Chr03B:673845884-673846998 | - | 1027 | 1451 | 341 |
| C3 | *Triticum aestivum* | *TaTHI1-3* | *TraesCS7B02G278200* | PLAZA Monocot 4.5 | Chr07B:509512400-509513581 | + | 1055 | 1716 | 351 |
| C3 | *Triticum aestivum* | *TaTHI1-4* | *TraesCS7D02G373100* | PLAZA Monocot 4.5 | Chr07D:483877570-483878752 | + | 1066 | 1787 | 354 |
| C3 | *Triticum turgidum* | *TtTHI1-1* | *TRITD7Av1G203210* | PLAZA Monocot 4.5 | Chr7A:546621905-546623236 | + | 1027 | 1126 | 341 |
| C3 | *Triticum turgidum* | *TtTHI1-2* | *TRITD3Bv1G222500* | PLAZA Monocot 4.5 | Chr3B:677362538-677363652 | - | 1027 | 1126 | 341 |
| C3 | *Triticum turgidum* | *TtTHI1-3* | *TRITD7Bv1G156750* | PLAZA Monocot 4.5 | Chr7B:493495850-493497031 | + | 1066 | 1193 | 354 |
| C4 | *Cenchrus americanus* | *CaTHI1-1* | *Pgl_GLEAN_10010164* | PLAZA Monocot 4.5 | Chr07:224477829-224478779 | - | 857 | 960 | 284 |
| C4 | *Cenchrus americanus* | *CaTHI1-2* | *Pgl_GLEAN_10003958* | PLAZA Monocot 4.5 | Chr05:148022397-148023524 | + | 1030 | 1139 | 342 |
| C4 | *Eleusine coracana* | *EcTHI1-1* | *ELECO.r07.7AG0569970.1* | Phytozome 13 | Chr07A:36147479-36148675 | + | 1071 | 1196 | 357 |
| C4 | *Eleusine coracana* | *EcTHI1-2* | *ELECO.r07.7BG0601740.1* | Phytozome 13 | Chr07B:49133702-49134896 | + | 1071 | 1194 | 357 |
| C4 | *Miscanthus sinensis* | *MsTHI1-1* | *Misin05G173500* | PLAZA Monocot 4.5 | Chr05:58519175-58520333 | + | 1072 | 1170 | 356 |
| C4 | *Miscanthus sinensis* | *MsTHI1-2* | *Misin06G165200* | *PLAZA Monocot 4.5* | *Chr06:60309162-60310319* | *-* | *1072* | *1169* | *356* |
| C4 | *Miscanthus sinensis* | *MsTHI1-3* | *Misin04G366400* | *PLAZA Monocot 4.5* | *Chr04:108217061-108218194* | *-* | *1069* | *1145* | *355* |
| C4 | *Miscanthus sinensis* | *MsTHI1-4* | *MisinT322000* | *PLAZA Monocot 4.5* | *scaffold00815 : 47844-48980* | *+* | *1062* | *1137* | *353* |
| C4 | *Panicum halli var. hallii* | *PhTHI1* | *Pahal.4G068300* | *Phytozome 13* | *Chr04:4505030-4507290* | *-* | *1053* | *1794* | *351* |
| C4 | *Panicum virgatum* | *PvTHI1-1* | *Pavir.4NG251200* | *Phytozome 13* | *Chr04N:43061689-43063378* | *+* | *1053* | *1689* | *351* |
| C4 | *Panicum virgatum* | *PvTHI1-2* | *Pavir.4KG345900* | *Phytozome 13* | *Chr04K:41029354-41031130* | *+* | *1059* | *1776* | *353* |
| C4 | *Saccharum* sp. var. R570 | *SHRBa-017_B18* | *SHCRBa_017_B18* | *-* | *SHCRBa_017_B18* | *+* | *1059* | *1182* | *359* |
| C4 | *Saccharum* sp. var. R570 | *SHRBa-094_O04* | *SHCRBa_094_O04* | *-* | *SHCRBa_094_O04* | *+* | *1056* | 1170 | 356 |
| C4 | *Saccharum* sp. var. R570 | *SHRBa-108_C04* | *SHCRBa_108_C04* | *-* | *SHCRBa_108_C04* | *+* | *1068* | 1170 | *356* |
| C4 | *Saccharum spontaneum* | *SsTHI1-1* | *Sspon.03G0013100-1A* | *PLAZA Monocot 4.5* | *Chr3A:36535607-36536757* | *+* | *1071* | *2390* | *356* |
| C4 | *Saccharum spontaneum* | *SsTHI1-2* | *Sspon.03G0013100-2B* | *PLAZA Monocot 4.5* | *Chr3B:46285649-46286799* | *+* | *1070* | *2390* | *356* |
| C4 | *Saccharum spontaneum* | *SsTHI1-3* | *Sspon.02G0003080-1A* | *PLAZA Monocot 4.5* | *Chr2A:10117268-10118398* | *+* | *1041* | *2304* | *347* |
| C4 | *Saccharum spontaneum* | *SsTHI1-4* | *Sspon.02G0003080-2C* | *PLAZA Monocot 4.5* | *Chr2C:9257115-9258218* | *+* | *1041* | *1104* | *347* |
| C4 | *Setaria italica* | *SiTHI1-1* | *Seita.2G337800* | *Phytozome 13* | scaffold02:42177038-42178616 | + | 1056 | 2090 | 352 |
| C4 | *Setaria italica* | *SiTHI1-2* | *Seita.4G199600* | *Phytozome 13* | scaffold04:31839872-31841962 | + | 1173 | 1578 | 391 |
| C4 | *Setaria viridis* | *SvTHI1-1* | *Sevir.2G348100* | *Phytozome 13* | Chr02:40998754-41000650 | + | 1053 | 1896 | 351 |
| C4 | *Setaria viridis* | *SvTHI1-2* | *Sevir.4G208400* | *Phytozome 13* | Chr04:31067862-31069403 | + | 1056 | 1541 | 352 |
| C4 | *Sorghum bicolor* | *SbTHI1-1* | *Sobic.003G191000* | *Phytozome 13* | Chr03:51236001-51237593 | - | 1065 | 1592 | 355 |
| C4 | *Sorghum bicolor* | *SbTHI1-2* | *Sobic.002G384400* | *Phytozome 13* | Chr02:73966176-73967637 | - | 1059 | 1461 | 353 |
| C4 | *Zea mays* | *ZmTHI1-1* | *Zm00001d011183* | *Phytozome 13* | Chr08:142217717-142219220 | - | 1065 | 1503 | 355 |
| C4 | *Zea mays* | *ZmTHI1-2* | *Zm00001d044228* | *Phytozome 13* | Chr03:222461983-222463639 | - | 1068 | 1656 | 356 |
