# Supplementary material for: Functional and comparative analysis of THI1 gene in grasses with a focus on sugarcane
Source: PeerJ. 2023 May 15;11:e14973. doi: 10.7717/peerj.14973 (PMC10194071; doi:10.7717/peerj.14973)
Supplement: Supplemental Information 6 — Total number and number related to each ScTHI1 copy. [file peerj-11-14973-s006.docx]

**Table S4. Clones assembled from each genome**. Total number and number related to each *ScTHI1* copy.

| **Cultivar name** | **Number of clones to each *ScTHI1* copy** | | **Total of clones by cultivar** |
| --- | --- | --- | --- |
|  | ***ScTHI1-1*** | ***ScTHI1-2*** |  |
| R570 (BACs) | 9 | 10 | 19 |
| *Miscanthus sp.* | 8 | 3 | 11 |
| *S. spontaneum* | 9 | 9 | 18 |
| *S. spontaneum (IN84-58)* | 7 | 5 | 12 |
| *S. officinarum* | 6 | 2 | 8 |
| Co-290 | 10 | 6 | 16 |
| NA56-79 | 7 | 3 | 10 |
| Nco-310 | 7 | 2 | 9 |
| POJ-2878 | 15 | 2 | 17 |
| RB72454 | 10 | 7 | 17 |
| RB835486 | 8 | 9 | 17 |
| RB867515 | 8 | 2 | 10 |
| SP70-1143 | 3 | 8 | 11 |
| SP80-3280 | 9 | 3 | 12 |
| SP81-3250 | 2 | 8 | 10 |
| **Total** | **118** | **79** | **197** |
